# Are Healthcare Providers Asking about Environmental Exposures? A Community-Based Mixed Methods Study

**DOI:** 10.1155/2015/189526

**Published:** 2015-10-08

**Authors:** Kristina M. Zierold, Clara G. Sears

**Affiliations:** Department of Epidemiology and Population Health, School of Public Health, University of Louisville, 485 East Gray Street, Louisville, KY 40202, USA

## Abstract

People living near environmental hazards may develop symptoms and health conditions that require specialized monitoring and treatment by healthcare providers. One emerging environmental hazard is coal ash. Coal ash is comprised of small particles containing heavy metals, polycyclic aromatic hydrocarbons, and radioactive elements. The overall purpose of this study was to explore whether healthcare providers ask patients if they live near an environmental hazard like coal ash storage sites and to assess what health conditions prompt a provider inquiry. Focus groups were conducted in 2012 and a cross-sectional survey was administered in 2013. Overall, 61% of survey respondents reported that their healthcare providers never asked if they lived near an environmental hazard. One focus group member stated “No, they don't ask that. They just always blame stuff on you….” Respondents with asthma and other lung conditions were significantly more likely to be asked by a healthcare provider if they lived near an environmental hazard. Due to the unique exposures from environmental hazards and the low prevalence of patients being asked about environmental hazards, we recommend that healthcare providers take environmental health histories in order to understand patients' exposures, to monitor symptoms of exposure, and to assist with education about reducing exposure.

## 1. Introduction

People who reside near environmental hazards face unique exposures that complicate health. Due to the potentially chronic exposures associated with living near environmental hazards, healthcare providers need to know and understand their patient's exposures in order to monitor symptoms of exposure, provide appropriate treatment of health conditions, and assist with education about reducing exposure. Asking patients if they live near environmental hazards and taking an environmental health history should be a standard component of a patient's health history.

There are limited studies conducted among healthcare providers regarding training in environmental health. Trasande et al. [1–4] found that healthcare providers who care for children report that they never receive training in environmental health history taking and have limited training in environmental health, and many lack knowledge about environmental exposures. Trasande et al. [4] reported that providers were comfortable dealing with lead and second-hand smoke, but confidence in managing other pollutants, including air pollution, mercury, pesticides, and mold, was much lower.

In adult patients, Stotland et al. [5] found that only 1 in 15 obstetricians received training in environmental health. In a survey of 2,514 obstetricians, less than 20% reported routinely asking about environmental exposures commonly found in pregnant women in the United States. Furthermore, 50% reported that they rarely take an environmental health history. Hamilton et al. [6] surveyed 350 primary care physicians and found that 86% reported no specific training in environmental health history taking. These findings are alarming considering that millions of people reside near environmental hazards which can be complex mixtures of pollutants.

One growing environmental hazard of concern in the United States is coal ash. Coal ash, a waste product generated from burning coal for electricity, is a significant public health concern. In 2013, the American Coal Ash Association [7] reported that coal-fired power plants were responsible for producing 114 million tons of coal ash, making it one of the largest waste streams in the US. The toxicity of ash varies; however, Brown et al. [8], El-Mogazi et al. [9], Gottlieb et al. [10], Papastefanou [11], and the Research Triangle Institute [12] found that coal ash frequently contains heavy metals, polycyclic aromatic hydrocarbons, and radioactive elements.

Brown et al. [8] and Jones et al. [13] report that the dominant component of coal ash is fly ash, which consists of small, spherical particles with diameters predominately ≤10 *μ*m. These small particles have the potential for being highly hazardous, as they have the ability to penetrate deep into the lungs and enter the bloodstream. Jones et al. [13] found that approximately 60% of coal ash is stored in piles, landfills, or surface impoundments, which allow for fugitive dust emissions and infiltration into ground water. The United States Environmental Protection Agency (US EPA) [14, 15] estimates that there are approximately 300 landfills and 584 ash ponds throughout the US, although the actual number may be much greater. The US EPA [15] estimates that over 6 million people, including 1.5 million children, are exposed to coal ash.

Research investigating whether healthcare providers ask about environmental exposures has been conducted from the perspective of healthcare providers; yet no research has explored the community's experience. Therefore, working with a community chronically exposed to coal ash, this study assessed from the community's perspective if healthcare providers ask about environmental exposures. This research had three objectives: (1) to explore if healthcare providers ask patients whether they reside near a coal ash storage site, (2) to evaluate if differences in demographics and perception of health are related to whether or not a healthcare provider asks about living near a coal ash storage site, and (3) to assess which health conditions were associated with prompting a healthcare provider to ask if a patient lived near an environmental hazard.

## 2. Methods

From 2011 to 2013, a community-based mixed methods study was conducted with neighborhoods adjacent to a large coal ash storage site in Kentucky. The population is predominately low-income, multigenerational, nontransient, and white. The coal ash storage site has been a concern of the community for years, with community members claiming that ash blows throughout the neighborhoods.

Focus groups were conducted during summer 2012 and a cross-sectional survey was administered in spring/summer 2013. All procedures were approved by the University of Louisville Institutional Review Board and consent was obtained from all participants.

### 2.1. Focus Groups

In summer 2012, five focus groups with 26 adults were conducted. All focus group participants lived in one of four neighborhoods adjacent to the coal ash storage site. Detailed methods for the focus groups have been published by Zierold and Sears [16]. In brief, in June 2012, two community-wide meetings were held to explain the project and answer questions from the community. Community members were notified of the meetings by flyers that were distributed in the neighborhoods and by community leaders who lived in the neighborhoods. At the community-wide meeting, community members who were interested in participating in a focus group provided their names, addresses, and phone numbers so that they could be scheduled for a focus group in July or August.

Focus groups were held on Wednesday evenings in a private room at a restaurant located near the neighborhoods. A semistructured guide that contained three sections, (1) community strengths and weaknesses, (2) perceptions and beliefs about coal ash and exposure, and (3) perceptions about community health and personal/family health, was used for the focus groups. Most participants spoke freely and prompts were not needed. All discussions were tape recorded and later transcribed verbatim by a medical transcriptionist. Since there are no studies on community populations exposed to coal ash, inductive thematic analysis based on work of Braun and Clarke [17] and Patton [18] was used to analyze the data from the transcripts.

Among the focus group participants, 16 (62%) were female and all were white. The average age of all participants was 51.3 years (SD = 13.9). Males were slightly older than females (55.5 years old (SD = 10.1) versus 48.4 years old (SD = 15.3; *p* < 0.05)). The majority of participants (75%) lived near the power plant for more than 15 years. Eighty-eight percent of adults reported having at least one health condition.

### 2.2. Questionnaire Design

Based on the themes identified from the focus groups, a questionnaire was designed to take into consideration the population's unique exposure. The questionnaire contained 39 questions that concentrated on coal ash exposure, health conditions, and health behaviors, such as smoking and wearing personal protective equipment when cleaning. Thirty-eight of the questions were multiple choice, and the final question was open-ended that was asked to respondents to describe how they knew they were exposed to coal ash. Before the questionnaire was administered to the community, the study team met with community leaders to pretest and discuss it. Some questions were revised based on feedback from the leaders. The final questionnaire was administered in May-June 2013 and August-September 2013. The questionnaire, which was five pages in length, took approximately 20–30 minutes to complete.

A total of 231 community members participated in the survey. The respondents were 53% female and the median age was 52 years old (IQR = 60–24). There was not a significant difference in median age between males (51 years old, IQR = 58–34) and females (53 years old, IQR = 62–37) (*p* = 0.229). Most (74%) owned their home and 49% lived in their neighborhood for more than 20 years.

### 2.3. Variables Used in Survey Analysis

The variables used for analysis are broken down into four characteristics: demographics, perceptions of health, health conditions, and healthcare provider inquiry. Demographics included gender, current age, length of time living near the coal ash storage site, and whether the respondent owned or rented their home. Information about smoking history, including whether community members were current smokers, past smokers, or nonsmokers, was also collected. To understand how community members perceived their health, two questions were used: “How would you describe your overall health (Excellent, Very good, Good, Fair, Poor)” and “I am as healthy as other people I know (True/False).”

To assess health conditions, the question “have you ever been told by a doctor or health care provider that you have (circle Y if Yes)…” was used. Thirty health conditions were given from which respondents could choose. The thirty health conditions that were used in the questionnaire were chosen based on the conditions that were mentioned frequently during the focus groups and were highlighted as chronic problems in the community. Data is only reported for health conditions with a prevalence of 10% or greater. Health conditions reported on the survey were not verified by healthcare personnel. However, research conducted by Bush et al. [19], Haapanen et al. [20], Kriegsman et al. [21], Mukerji et al. [22], and Skinner et al. [23] found that among adult populations self-report is well validated and that there is a high agreement between self-report and medical report for many symptoms and conditions.

To assess if healthcare providers inquired about residents living near an environmental hazard, the question “has a doctor or health care provider ever asked if you lived near an environmental hazard (Y/N/I don't know)” was used. For the analysis, participants who answered “I don't know” were removed.

### 2.4. Statistical Analysis

Data was analyzed using SAS 9.3 (NC). Frequencies and counts were done to determine the percent of healthcare providers asking and not asking by demographics and health conditions. The distribution of age was not normal based on the Shapiro-Wilk statistic, and therefore medians and interquartile ranges (IQR) are presented. The Wilcoxon Rank Sum Test was used to evaluate the differences between medians.

For demographic variables, health perceptions, and health conditions, comparisons between healthcare providers asking and not asking were done using chi-square analysis and Fisher's Exact Test, which is appropriate for data with expected cell counts ≤5. Logistic regression was used to evaluate which health conditions were associated with prompting a healthcare provider to ask if a patient lived near a coal ash storage site.

## 3. Results

### 3.1. Focus Group Findings

Two themes regarding healthcare providers were identified: (1) lack of communication and (2) lack of awareness. None of the focus group participants were asked by their healthcare providers if they lived near an environmental hazard. One female respondent stated “no, they don't ask that. They just always blame stuff on you, on the food you eat and all that stuff.” Another female with children responded with the following:

*The only thing mine has ever asked is with the lead in the paint and stuff. No,  “Do you live around a coal ash plant? Have you lived…? Dah, dah, dah, a certain power plant,”  and stuff like that. No, they don't ask questions.*

This lack of communication also existed from patient to healthcare provider, as most never told their healthcare providers they lived near coal ash. One woman said the following: “No, to be honest with you I've never even thought about it. I never have.” Even a woman suffering from chronic laryngitis stated the following: “I'm going to have to say I haven't either….”

A second theme identified was lack of awareness about coal ash among healthcare providers. Many participants believed that the reason they were never asked about exposure to coal ash was because their healthcare providers were not aware of coal ash. One female participant stated and others agreed on the following:

*When they go to the doctors and stuff like that, and you think,  “Does your doctor ask you,  ‘Do you live on a coal ash plant?'” See, that's what I'm saying. Doctors really don't know because they don't know*

Participant #2:* “Right. They don't know. They're not aware.”*

Others claimed that they were treated for conditions and/or situations that were not accurate. Yet others stated that healthcare providers could not figure out what was causing the problem. One woman shared the following about her adult daughter: “She's a nurse. She gets them {headaches}. She says it's unbearable at time… It's miserable. I've watched her. They hurt so bad. She gets sick to her stomach. She's been to neurologist, on top of neurologist, on top of neurologist. None of them could figure out what the problem is.”

### 3.2. Cross-Sectional Survey Findings

Of the survey respondents, 61% reported that their healthcare providers never asked if they lived near an environmental hazard, 12% did not know if they had been asked, and only 27% were asked by a healthcare provider.  Table 1 reports the demographics and health perception comparisons between patients who were asked and those who were not asked by their healthcare providers. There were no differences by patient's age, gender, property ownership, length of time living near the coal ash storage site, or smoking status. However, a borderline significant difference existed in the percent of patients who perceived they were in poorer health, compared to others (*p* = 0.059). A significant difference was found between a healthcare provider asking and a healthcare provider not asking among people who felt they were not as healthy as other people (*p* < 0.001).

Table 2 lists the health conditions and the percent of patients that were asked about living near an environmental hazard. The five most prevalent health conditions were allergies (54%), high blood pressure (43%), anxiety (34%), depression (32%), and asthma (26%). Patients with allergies, asthma, and other lung conditions were significantly more likely to be asked if they lived near an environmental hazard.

Table 3 reports the results of the logistic regression to assess which health conditions were more likely to prompt a healthcare provider to ask a patient about living near an environmental hazard. Allergies (AOR = 2.05, 95% CI = 1.08–3.88), asthma (AOR = 2.49, 95% CI = 1.26–4.91), and other lung conditions (AOR = 2.47, 95% CI = 1.15–5.32) were associated with healthcare providers asking patients if they lived near an environmental hazard.

## 4. Discussion

This study highlighted that overall very few healthcare providers inquire about living near environmental hazards, like coal ash storage sites. Only 27% of community members were asked by their healthcare providers if they lived near an environmental hazard. Due to the unique and potentially chronic exposures from environmental hazards, healthcare providers would benefit from understanding patients' exposure in order to monitor symptoms of exposure and to assist with education about reducing exposure. Why healthcare providers did not ask community members about living near a coal ash storage site is unknown. They may not be aware of coal ash as many focus group participants believed, or healthcare providers may know about coal ash and already have considered it when treating the patient. The intent of this study was to document the community's experience; however, future work documenting healthcare providers' experience would be useful in understanding the provider-patient relationship.

Three health conditions (allergies, asthma, and other lung conditions) were likely to elicit a healthcare provider asking about living near an environmental hazard. These conditions could be caused by or exacerbated by exposure to small particles, such as coal ash. However, in many cases, such as nerve disease (metals exposure), heart disease (particulate exposure), depression (heavy metals), or hearing loss (heavy metals), healthcare providers did not ask. Many of the health conditions that patients experience from environmental exposures are common conditions, particularly among middle-aged and older adults, so healthcare providers may not recognize that the health conditions may be due to environmental causes. This is especially true if no environmental health history was taken. This study took place 20 miles from a large city with numerous hospitals and healthcare facilities and in which other environmental hazards are located, yet focus group participants and survey respondents were not asked about living near environmental hazards. If healthcare providers do not ask patients if they live near environmental hazards, how can providers communicate environmental health risks and exposure reduction strategies that adequately treat and manage patients' health conditions?

Healthcare providers, especially those working with low-income communities where environmental hazards are likely to exist, need better training regarding environmental health. Environmental health history should be a standard component of the health history of every patient. Previous studies with pediatricians found that most did not receive training in environmental history taking and had limited ability to manage diseases of environmental origin. Most limit their inquiry to tobacco smoke and lead [1–4, 6]. Limited studies among practitioners who care for adults report similar patterns: no training in environmental health history taking and not asking about environmental exposures [5, 6].

Our findings provide evidence from a patient's perspective of the breakdown in communication about environmental health that could provide insight for healthcare providers. Focus group results suggest that healthcare providers are likely to first attribute health conditions to an individual's behaviors or actions (i.e., diet, alcohol consumption, and exercise) without considering the context (environment) that may be acting on the patient. Making healthcare providers aware of environmental hazards surrounding the communities they service and training them to consider health behaviors within the context of a patient's environment may increase the likelihood that patients and providers will have a conversation about a variety of environmental exposures.

Communication is a two-way street and healthcare involves a relationship between the provider and the patient. Focus group results found that patients were unlikely to speak up and say they lived near a coal ash storage site when visiting a healthcare provider, even when the provider was unable to determine the cause of the health problem. Patients need to be encouraged to recognize and discuss all concerns related to hazardous environmental exposures near their homes.

### 4.1. Limitations

There are a few limitations of this study which need to be considered. First, although all members of the community were encouraged to participate in this study, the members that did participate might not be representative of the entire community. The sample may be more knowledgeable about coal ash or may be more affected by coal ash or have less fear about retribution from the company. However, the population is fairly homogeneous and focus groups and anecdotal evidence suggests that the majority of community members suffer symptoms or health conditions related to coal ash exposure. A second limitation of this study is that it might not be generalizable to other communities because we recruited participants who lived near a large coal ash storage site in Kentucky. Finally, this study did not discern between different healthcare providers; so we cannot determine or contrast which providers were more likely to inquire about environmental hazards. Additional research that overcomes these limitations is needed. Studies that involve populations from multiple states will allow the findings to be generalizable. To improve upon the understanding of healthcare providers who do and do not ask about living near environmental hazards, more detailed information about the providers should be collected.

## 5. Conclusions

This study highlights that healthcare providers do not routinely ask about living near environmental hazards, like coal ash storage sites. Unlike other studies which focused on physicians, this study presents the community's experience with healthcare providers. Healthcare providers and patients need to better communicate information regarding living conditions so that treatment and education can take into account potential exposures from environmental hazards. Environmental health history should be part of every patient's health history, so that healthcare providers can provide adequate treatment.

## Figures and Tables

**Table 1:** Comparison of demographic and health perceptions of coal ash community members, by healthcare provider inquiry (*N* = 204)^a^.

Characteristic	Healthcare provider asked (*n* = 62)	Healthcare provider did not ask (*n* = 142)	*p* value
Gender			0.841
Female	35 (56%)	78 (55%)	
Male	27 (44%)	64 (45%)	
Median age	53 (IQR = 60–47)	51.5 (IQR = 58–34)	0.461^c^
Length of time at current home			
Less than 5 years	13 (21%)	28 (20%)	0.447^b^
5–10 years	7 (11%)	15 (11%)	
11–15 years	5 (8%)	16 (11%)	
16–20 years	3 (5%)	18 (13%)	
More than 20 years	34 (55%)	65 (46%)	
Property ownership^d^			0.969
Rent	16 (26%)	35 (25%)
Own	46 (74%)	102 (72%)
Current smoker^d^			0.121
Yes	16 (26%)	54 (38%)	
No	44 (71%)	88 (62%)	
Past smoker^d^			0.431
Yes	37 (60%)	93 (65%)	
No	24 (39%)	47 (33%)	
Description of overall health^d^			0.059^b^
Excellent	0	2 (1%)	
Very good	4 (6%)	18 (13%)	
Good	20 (32%)	64 (45%)	
Fair	21 (34%)	39 (27%)	
Poor	16 (26%)	18 (13%)	
I am as healthy as other people I know^d^			
True	15 (24%)	81 (57%)	<0.001^*∗∗∗*^
False	47 (76%)	56 (39%)	

^a^From the original sample (*N* = 231), 27 respondents answered “I don't know” to the healthcare provider question and were removed.

^b^Fisher's Exact Test.

^c^Wilcoxon Rank Sum Test with the normal approximation.

^d^Percents may not add to 100% due to missing responses.

^*∗∗∗*^Significant at *p* < 0.001.

**Table 2:** Health conditions of survey respondents by healthcare provider inquiry (*N* = 204)^a^.

Health condition (prevalence)	Healthcare provider asked (*n* = 62)	Healthcare provider did not ask (*n* = 142)	*p* value
Allergies (54%)			0.044^*∗*^
Yes	40 (65%)	70 (49%)	
No	22 (35%)	72 (51%)	
High blood pressure (43%)			0.700
Yes	28 (45%)	60 (42%)	
No	34 (55%)	82 (58%)	
Anxiety (34%)			0.130
Yes	26 (42%)	44 (31%)	
No	36 (58%)	98 (69%)	
Depression (32%)			0.985
Yes	20 (32%)	46 (32%)	
No	42 (68%)	96 (68%)	
Asthma (26%)			0.017^*∗*^
Yes	23 (37%)	30 (21%)	
No	39 (63%)	112 (79%)	
Other lung diseases (17%)			0.030^*∗*^
Yes	16 (26%)	19 (13%)	
No	46 (74%)	123 (87%)	
Hearing loss (15%)			0.215
Yes	12 (19%)	18 (13%)	
No	50 (81%)	124 (87%)	
Heart disease (13%)			0.088
Yes	12 (19%)	15 (11%)	
No	50 (81%)	127 (89%)	
Cancer (13%)			0.420
Yes	10 (16%)	17 (12%)	
No	52 (84%)	125 (88%)	
Kidney problems (13%)			0.338
Yes	10 (16%)	16 (11%)	
No	52 (84%)	126 (89%)	
Nerve disease (13%)			0.157
Yes	11 (18%)	15 (11%)	
No	51 (82%)	127 (89%)	
Type 2 diabetes (11%)			0.472^b^
Yes	5 (8%)	17 (12%)	
No	57 (92%)	125 (88%)	
Thyroid disease (11%)			0.996
Yes	7 (11%)	16 (11%)	
No	55 (89%)	126 (89%)	
Anemia (10%)			0.968
Yes	6 (10%)	14 (10%)	
No	56 (90%)	128 (90%)	

^a^From the original sample (*N* = 231), 27 respondents answered “I don't know” to the healthcare provider question and were removed.

^b^Fisher's Exact Test.

^*∗*^Significant at *p* < 0.05.

**Table 3:** Conditions associated with healthcare providers asking about living near a coal ash storage site.

Health condition (prevalence)	Crude OR, 95% CI	Adjusted OR^a^, 95% CI
Allergies (54%)	1.87 (1.01–3.46)^*∗*^	2.05 (1.08–3.88)^*∗*^
High blood pressure (43%)	1.13 (0.62–2.05)	1.16 (0.63–2.14)
Anxiety (34%)	1.61 (0.87–2.98)	1.69 (0.90–3.18)
Depression (32%)	0.99 (0.53–1.89)	1.04 (0.54–1.98)
Asthma (26%)	2.02 (1.15–4.24)^*∗*^	2.49 (1.26–4.91)^*∗*^
Other lung conditions (17%)	2.25 (1.07–4.75)^*∗*^	2.47 (1.15–5.32)^*∗*^
Hearing loss (15%)	1.65 (0.74–3.68)	1.91 (0.82–4.43)
Heart disease (13%)	2.03 (0.89–4.65)	2.15 (0.92–5.03)
Cancer (13%)	1.41 (0.61–3.29)	1.42 (0.60–3.40)
Kidney problems (13%)	1.52 (0.65–3.56)	1.51 (0.63–3.64)
Nerve disease (13%)	1.83 (0.79–4.24)	1.88 (0.80–4.44)
Type 2 diabetes (11%)	0.65 (0.23–1.84)	0.72 (0.25–2.11)
Thyroid disease (11%)	1.00 (0.39–2.57)	1.03 (0.40–2.68)
Anemia (10%)	0.98 (0.36–2.68)	1.01 (0.36–2.82)

^a^Adjusted for length of time living near a coal ash storage site.

^*∗*^Significant at *p* = 0.05.
